# Production of Polyhydroxyalkanoates Using Hydrolyzates of Spruce Sawdust: Comparison of Hydrolyzates Detoxification by Application of Overliming, Active Carbon, and Lignite

**DOI:** 10.3390/bioengineering4020053

**Published:** 2017-05-28

**Authors:** Dan Kucera, Pavla Benesova, Peter Ladicky, Miloslav Pekar, Petr Sedlacek, Stanislav Obruca

**Affiliations:** Faculty of Chemistry, Brno University of Technology, Purkynova 118, 612 00 Brno, Czech Republic; Dan.Kucera@vut.cz (D.K.); pavla.benesova@vut.cz (P.B.); peter.ladicky@vut.cz (P.L.); pekar@fch.vut.cz (M.P.); sedlacek-p@fch.vut.cz (P.S.)

**Keywords:** polyhydroxyalkanoates, detoxification, lignite, *Burkholderia*

## Abstract

Polyhydroxyalkanoates (PHAs) are bacterial polyesters which are considered biodegradable alternatives to petrochemical plastics. PHAs have a wide range of potential applications, however, the production cost of this bioplastic is several times higher. A major percentage of the final cost is represented by the price of the carbon source used in the fermentation. *Burkholderia cepacia* and *Burkholderia sacchari* are generally considered promising candidates for PHA production from lignocellulosic hydrolyzates. The wood waste biomass has been subjected to hydrolysis. The resulting hydrolyzate contained a sufficient amount of fermentable sugars. Growth experiments indicated a strong inhibition by the wood hydrolyzate. Over-liming and activated carbon as an adsorbent of inhibitors were employed for detoxification. All methods of detoxification had a positive influence on the growth of biomass and PHB production. Furthermore, lignite was identified as a promising alternative sorbent which can be used for detoxification of lignocellulose hydrolyzates. Detoxification using lignite instead of activated carbon had lower inhibitor removal efficiency, but greater positive impact on growth of the bacterial culture and overall PHA productivity. Moreover, lignite is a significantly less expensive adsorbent in comparison with activated charcoal and; moreover, used lignite can be simply utilized as a fuel to, at least partially, cover heat and energetic demands of fermentation, which should improve the economic feasibility of the process.

## 1. Introduction

Polyhydroxyalkanoates (PHAs) are polyesters which are synthesized by numerous naturally occurring microorganisms as energy and carbon storage materials. Moreover, due to their mechanical and technological properties resembling those of some petrochemical plastics, PHAs are generally considered a biodegradable alternative to petrochemical-based synthetic polymers [1]. PHAs have a wide range of potential applications, however, the production cost of these bioplastics are several times higher which complicates their production at an industrial scale [2].

A substantial percentage of the final cost is represented by price of carbon substrate [3]. This is the motivation for seeking alternative sources for PHAs production. Among numerous inexpensive or even waste substrates, lignocellulose materials—with sn annual generation of 80 billion tons—represent one of the most promising resources for biotechnological production of (not only) PHAs [4]. Nevertheless, utilization of lignocellulosic materials is accompanied by numerous obstacles stemming from the complex nature of these materials. To access fermentable sugars from cellulose and hemicellulose, a hydrolytic step is required. The hydrolysis of complex lignocellulose biomass is usually performed in two steps. Diluted mineral acid is used in the first step to hydrolyze hemicellulose and to disrupt the complex structure of lignocellulose, which enables subsequent enzymatic hydrolysis of cellulose [5]. Nevertheless, apart from utilizable sugars, also numerous microbial inhibitors such as organic acids (e.g., acetic, formic or levulinic acid), furfurals, and polyphenols are generated by the hydrolysis process. These substances usually reduce fermentability of the hydrolyzates and decrease yields of the biotechnological processes. This problem can be solved by introduction of detoxification. Generally, the aim of detoxification is to selectively remove or eliminate microbial inhibitors from the hydrolyzate prior to biotechnological conversion of the hydrolyzate into desired products [6].

Numerous detoxification methods are based on more or less selective removal of inhibitors by their adsorption on various sorbents. The most commonly used sorbent for this purpose is active carbon, nevertheless, this detoxification strategy suffers from high cost of the sorbent [6]. On the contrary, lignite represents a very promising, low-cost, and effective sorbent which has already been used for the treatment of wastewater to remove various organic and inorganic contaminants [7].

The woodworking industry generates a variety of solid waste materials, such as sawdust, shavings, or bark. It is true that many of these waste materials are already used in various applications. Wood waste is very often burned and used for heat and electricity generation. On the contrary, it could be a potentially inexpensive and renewable feedstock for biotechnological production of PHAs. For instance, Pan et al. employed *Burkholderia cepacia* for biotechnological production of PHAs from detoxified maple hemicellulosic hydrolyzate [8]. Further, Bowers et al. studied PHA production wood chips of *Pinus radiata* which were subjected to high-temperature mechanical pre-treatment or steam explosion in the presence of sulphur dioxide before being enzymatically hydrolyzed. *Novosphingobium nitrogenifigens* and *Sphingobium scionense* were used for PHA production on these hydrolyzates [9]. *Brevundimonas vesicularis* and *Sphingopyxis macrogoltabida* were employed by Silva et al. [10] to produce ter-polymer consisting of 3-hydroxybutyrate, 3-hydroxyvalerate, and lactic acid (3-hydroxypropionate) from acid hydrolyzed sawdust. Despite the fact that PHA production capabilities are exhibited by many bacterial strains, *Burkholderia cepacia* and *Burkholderia sacchari* are the most commonly used for PHA production from hydrolyzates of lignocellulosic materials [8,11,12].

In this study, wood hydrolyzate was utilized as a carbon source for production of polyhydroxyalkanoates. Moreover, since hydrolyzates contain substantial concentrations microbial inhibitors, various detoxification methods including the novel application of lignite as a sorbent are used to improve the fermentability of wood hydrolyzate based media and thus, the PHA yields obtained on this promising substrate.

## 2. Materials and Methods

### 2.1. Wood Hydrolyzate (WH) Preparation

Spruce sawdust was supplied by a wood processing company. The waste material was firstly dried to constant weight (80 °C for 24 h). Sawdust was then pretreated with diluted acid and thereafter subjected to enzymatic hydrolysis. To hydrolyze the hemicelluloses of raw material, 20% (*w*/*v*) pre-dried sawdust was treated by 4% H_2_SO_4_ for 60 min at 121 °C. Enzymatic hydrolysis, as a following step, was used for digestion of cellulose structure to release further fermentable saccharides. It was performed by adjusting the pH of the suspension to 5.0 by NaOH and cellulose was treated by 0.5% of Viscozyme L (Sigma-Aldrich, Deisenhofen, Germany) at 37 °C under permanent shaking for 24 h. Subsequently, solids were removed by filtration and the permeate, called wood hydrolyzate (WH), was used in the preparation of the cultivation medium and for PHA production.

### 2.2. Microorganisms and Cultivation

*Burkholderia cepacia* (CCM 2656) was purchased from Czech Collection of Microorganisms, Brno, Czech Republic. *Burkholderia sacchari* (DSM 17165) was purchased from Leibnitz Institute DSMZ-German Collection of Microorganism and Cell Cultures, Braunschweig, Germany. The mineral salt medium for *B. cepacia* and *B. sacchari* cultivation was composed of: 1 g L^−1^ (NH_4_)_2_SO_4_, 1.5 g L^−1^ KH_2_PO_4_, 9.02 g L^−1^ Na_2_HPO_4_·12H_2_O, 0.1 g L^−1^ CaCl_2_·2H_2_O, 0.2 g L^−1^ MgSO_4_·7H_2_O, and 1 mL L^−1^ of microelement solution, the composition of which was as follows: 0.1 g L^−1^ ZnSO_4_·7H_2_O, 0.03 g L^−1^ MnCl_2_·4H_2_O, 0.3 g L^−1^ H_3_BO_3_, 0.2 g L^−1^ CoCl_2_, 0.02 g L^−1^ CuSO_4_·7H_2_O, 0.02 g L^−1^ NiCl_2_·6H_2_O, 0.03 g L^−1^ Na_2_MoO_4_·2H_2_O. The cultivations were performed in Erlenmeyer flasks (volume 100 mL) containing 50 mL of the cultivation medium. The temperature was set to 30 °C and the agitation to 180 rpm. The cells were harvested after 72 h of cultivation.

### 2.3. Detoxification of Hydrolyzates

Overliming was carried out as described by Ranatunga et al. [13], whereupon pH of the hydrolyzate was adjusted to approx. pH 10.0 using solid calcium hydroxide. The samples were then kept at 50 °C for 30 min, the pH was adjusted back to 7, and the sample was subsequently filtered through filter paper.

Detoxification with activated charcoal was performed as described by Pan et al. [8]. Charcoal was added to hydrolyzate in the ratio 1:20 (*w*/*v*) and stirred for 1 h at 60 °C. Solid particles were removed by filtration. Furthermore, detoxification with lignite was performed similarly, finely milled lignite power (grain size of under 0.2 mm) from South Moravian Coalfield (the northern part of the Vienna basin in the Czech Republic) was used.

### 2.4. Analytical Methods

All analyses of hydrocarbons and furfural were performed with a Thermo Scientific UHPLC system–UltiMate 3000. REZEX-ROA column (150 × 4.6 mm, 5 μm; City, Phenomenex, Torrance, California, USA) was used for separation. The mobile phase was 5 mN H_2_SO_4_ at a flow rate of 0.5 mL per min. Xylose and other saccharides were detected using a refractive index detector (ERC RefractoMax 520). Acetate, levulinic acid and furfural were detected with a Diode Array Detector (DAD-3000) at 284 nm.

Total phenolics were determinated as described by Li et al. [14] with the Folin–Ciocalteu reagent (Sigma-Aldrich). Gallic acid was used for calibration and total phenolics were expressed as milligrams of gallic acid equivalents per liter of wood hydrolyzate.

### 2.5. PHA Extraction and Content Analysis

To determine biomass concentration and PHA content in cells, samples (10 mL) were centrifuged and the cells were washed with distilled water. The biomass concentration expressed as cell dry weight (CDW) was analyzed as reported previously [15]. PHA content of dried cells was analyzed by gas chromatography (Trace GC Ultra, Thermo Scientific, Waltham, Massachusetts, USA) as reported by Brandl et al. [16]. Commercially available P(3HB-co-3HV) (Sigma Aldrich) composed of 88 mol. % 3HB and 12 mol. % 3HV was used as a standard; benzoic acid (LachNer, Neratovice, Czech Republic) was used as an internal standard.

## 3. Results and Discussion

The composite formed by cellulose, hemicellulose, and lignin is responsible for the remarkable resistance against hydrolysis and enzymatic attack [17]. Generally, proper pre-treatment of lignocellulose prior to its enzymatic hydrolysis by cellulases significantly improves fermentable sugar yields [18]. The combination of diluted acid hydrolysis (1% H_2_SO_4_) and enzymatic digestion of cellulose was used for hydrolysis of spruce sawdust. This approach yielded liquid hydrolyzate of wood (WH) and its composition is shown in Table 1.

Glucose is formed by the cleavage of cellulose. Hemicelluloses can be hydrolyzed to yield molecules such as xylose, arabinose, mannose, galactose, and uronic acid [19]. The total concentration of sugars in WH was determined to be 14.9 g L^−1^ (by the hydrolysis of 50 g L^−1^ of spruce sawdust). The only identified saccharides in WH are xylose (10.4 g L^−1^) and glucose (4.5 g L^−1^). Unfortunately, WH contains high concentrations of inhibiting substances such as polyphenols (1205 mg L^−1^), furfural (52.0 mg L^−1^), acetic acid (0.53 mg L^−1^), and levulinic acid (9.9 mg L^−1^). Polyphenols are likely to be released from waste wood biomass during the partial degradation of lignin by acid hydrolysis. Furfural is formed by degradation of reducing sugars at high pressure and low pH. Levulinic acid, the degradation product of furfural or 5-hydroxymethylfurfural, is formed in the same manner. Acetic acid is probably formed by deesterification of acetylated wood components. Moreover, the amount of ash is significant, which is a consequence of the application of sulfuric acid and subsequent neutralization by NaOH. High concentrations of salts may theoretically cause the inhibition of bacterial growth due to the induction of osmotic stress. On the contrary, mild osmotic up-shock was reported to support PHB accumulation in *Cupriavdius necator* H16 [20,21].

**Figure 1:**
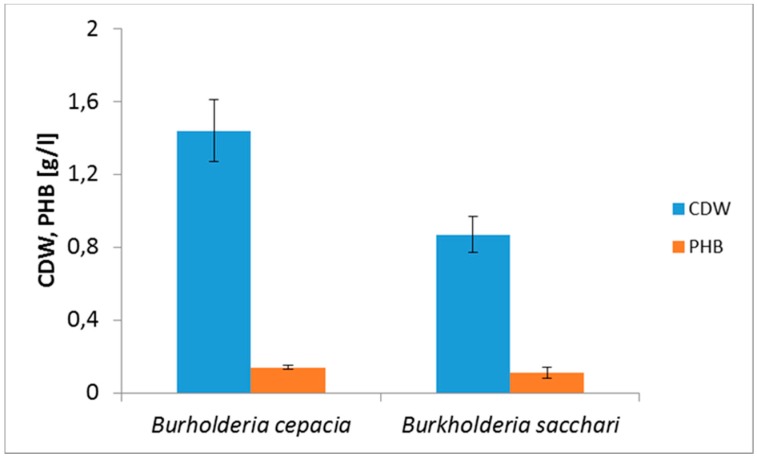
Cultivation of *B. cepacia* and *B. sacchari* on WH which composition is demonstrated in Table 1, WH was twice diluted and supplemented with mineral salts as described above. Cultivation conditions: 30 °C, 72 h, 180 rpm.

The hydrolyzate of waste wood biomass was used as the sole carbon source for PHA production employing *B. cepacia* and *B. sacchari*. Figure 1 demonstrates the negative impact of the presence of inhibitors on the intended biotechnological processes. In both cases, the WH was twice diluted prior to culturing and supplemented by mineral medium.

Yields of biomass were relatively low, approximately 1.0–1.5 g L^−1^, and PHB content in CDW was about 10%. Total yield of PHB was around 0.1 g L^−1^, which is very low.

The effect of phenolic and other aromatic compounds, which may inhibit both microbial growth and product yield, are very variable, and can be related to specific functional groups. One possible mechanism is that phenolics interfere with the cell membrane by influencing its function and changing its protein-to-lipid ratio [22]. Undissociated acids enter the cell through diffusion over the cell membrane and then dissociate due to the neutral cytosolic pH. The dissociation of the acid leads to a decrease in the intracellular pH, which may cause cell death. This effect is promoted by furfural and 5-HMF which cause higher cell membrane permeation and disturb the proton gradient over the inner mitochondrial membrane which inhibits regeneration of ATP and eventually can lead to cellular death [23]. A different mechanism of action of growth inhibitors results in a stronger synergistic effect.

The presence of inhibitors, and especially polyphenols, in the wood hydrolyzate appears to be crucial for the intended biotechnological process. Therefore, we continued to focus on the elimination of microbial inhibitors. In the first phase, we compared two common detoxification procedures—separation by adsorption inhibitors on activated carbon and over-liming. Theoretically, overliming is effective due to precipitation or chemical destabilization of inhibitors [13] and activated charcoal could improve the fermentability of hydrolysate by absorbing phenolic compounds and other inhibitory substances [24].

**Figure 2:**
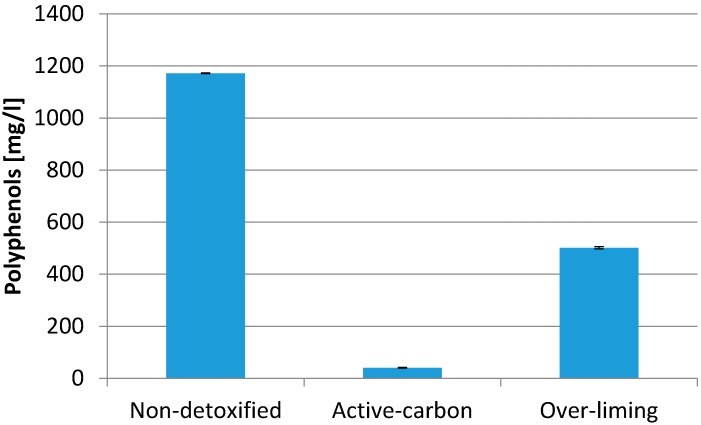
Detoxification employing over-liming and active carbon.

The effect of various methods of detoxification on the concentration of the most important inhibitors present in hydrolysates and polyphenols is demonstrated in Figure 2.

It is evident that both detoxification techniques significantly reduce the concentration of polyphenols in hydrolysates. More effective is the application of activated carbon, which can adsorb and thereby remove more than 90% of polyphenols. Table 2 demonstrates results of cultivation experiment with detoxified WH, employing the same PHB producers as in the previous test. Both methods of detoxification exhibited a positive influence on the growth of biomass, and this effect was more apparent with *B. cepacia*. More significantly, the effect of detoxification was reflected in the content of PHB in biomass. A positive effect on the biosynthesis of PHB occurred primarily in the strain of *B. sacchari*. PHB content reached nearly 90% of CDW. The yields were 8–12 times higher compared to the use of non-detoxified hydrolyzate. On the other hand, the detoxification process itself is time-consuming and particularly expensive, especially if activated carbon is used for detoxification of the hydrolyzates [25].

A further aim of our experiments was to find an alternative sorbent, which would be comparable to activated carbon, but the cost of which would be significantly lower. After several pilot experiments, we focused on lignite. It is the youngest and the least carbonized brown coal, which consists of a macromolecular complex polyelectrolyte (e.g., humic acids), polysaccharides, polyaromatics, and carbon chains with sulfur, nitrogen, and oxygen-containing groups. Its cost is significantly lower than that of activated carbon. The price of activated carbon is currently around $1/kg [26] compared to a lignite price of $0.2/kg [26]. Moreover, the recovery of activated charcoal after its application as a sorbent in detoxification is practically impossible [27,28]. On the other side, lignite can be burned after absorbing the inhibitors and the energy released during the combustion process could provide energy which can at least partially cover energetic demands of the intended process of PHA production from waste wood biomass.

The adsorption capacity of lignite and its application as a sorbent is often a subject of interest. It is the price of conventional sorbents that leads to finding low-cost alternatives [29]. Over the last decade, there has been an increase in publications dealing with low-cost adsorbents for wastewater treatment [30]. For instance, lignite was used as a sorbent for removal of organic substances such as phenol [31] or inorganic components, especially heavy metals [32], from contaminated water solutions. Nevertheless, to our best of our knowledge despite its high sorption capacity and low cost, lignite has not been used as a sorbent for detoxification of complex lignocellulose hydrolyzates to increase their fermentability and yield of biotechnological products.

According to our results, lignite has a lower sorption capacity than activated charcoal. On the other hand, lignite is also able to eliminate a substantial amount of inhibitors, and thus potentially increase the fermentability of WH. Comparison of lignite and activated charcoal as a sorbent for microbial inhibitors is displayed in Table 3.

**Figure 3:**
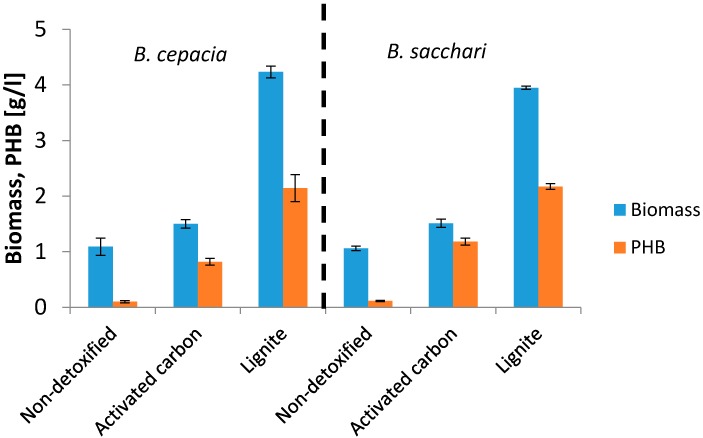
Cultivation on detoxified hydrolyzates.

The sorption properties of lignite depend on the number of sorption sites or functionalities [33]. Understandably, WH detoxified with lignite were also tested for cultivation. Detoxified hydrolyzates using lignite and activated carbon were used for the biotechnological production of PHB employing *B. cepacia* and *B. sacchari*. Figure 3 shows the results.

It is interesting that the sorption capacity of lignite as detoxification strategy does not provide as good a removal of monitored inhibitors as in the case of activated carbon. However, lignite is comparable with activated charcoal, considering the overall yields of PHB. *B. Sacchari* reached markedly higher yields. These surprisingly higher yields obtained by replacing active carbon with lignite should be explained and our further experiments will be focused in this direction. We assume that, during the detoxification, lignite released substances that had a positive effect on the growth of soil bacteria. Lignite is a complex material compared to activated carbon which contains only carbon. Therefore, use of lignite could have an enriching effect on the composition of the production medium. This interesting and surprising feature could also be used in other biotechnological processes in which soil originating microorganisms are employed.

## 4. Conclusions

This article demonstrates the possibilities of utilization of lignite in biotechnology. Lignite can be used as a sorbent to detoxify wood hydrolyzate and its efficiency was comparable with commonly used activated carbon. This detoxification method was evaluated directly using the hydrolyzates to produce PHAs employing *Burkholderia cepacia* and *Burkholderia sacchari*. The results showed that the use of lignite considerably improved fermentability of wood hydrolyzates and enhanced PHA yields. Therefore, lignite can cope with significantly more expensive activated carbon.

## Figures and Tables

**Table 1:** Composition of wood hydrolyzate (50 g of sawdust per 1 L of 4% H_2_SO_4_).

	Concentration
Glucose	4.5 g/L
Xylose	10.4 g/L
Ash	52,6 g/L
Polyphenols	1205 mg/L
Furfural	52.0 mg/L
Acetic acid	0.53 g/L
Levulinic acid	9.9 mg/L
5-HMF	*not detected*

**Table 2:** Cultivation on detoxified hydrolyzates.

	Detoxification	Biomass (g/L)	PHB (%)	PHB (g/L)
*Burkholderia sacchari*	Non-detoxified	0.87	12.2	0.11
	Over-liming	1.57	88.7	1.39
	Activated carbon	1.01	87.6	0.89
*Burkholderia cepacia*	Non-detoxified	1.44	9.8	0.14
	Over-liming	2.86	30.0	0.86
	Activated carbon	1.40	74.7	1.05

**Table 3:** Detoxification using active carbon and lignite

	Glucose (g/L)	Xylose (g/L)	Polyphenols (mg/L)	Furfural (mg/L)	Levulinic Acid (mg/L)	Acetic Acid (g/L)
Non-detoxified	4.4	10.0	998.8	41.4	10.0	0.5
Lignite	4.6	10.3	772.4	35.7	7.9	0.4
Activated carbon	4.5	10.1	23.8	3.8	3.6	0.4
